# The circadian genes are required in DAL neurons for *Drosophila* long-term memory formation

**DOI:** 10.3389/fnins.2025.1623251

**Published:** 2025-06-30

**Authors:** Jerry C. P. Yin, Ethan Cui, Peter S. Johnstone, Deniz Top, Grace Boekhoff-Falk, Hong Zhou

**Affiliations:** ^1^Laboratory of Genetics, School of Medicine and Public Health, University of Wisconsin-Madison, Madison, WI, United States; ^2^Neurology Department, School of Medicine and Public Health, University of Wisconsin-Madison, Madison, WI, United States; ^3^Department of Cell Biology, University of Alberta, Edmonton, AB, Canada; ^4^Department of Cell and Regenerative Biology, School of Medicine and Public Health, University of Wisconsin-Madison, Madison, WI, United States

**Keywords:** memory formation, circadian, *Drosophila*, DAL neurons, peripheral clock, mutants

## Abstract

We previously showed that memory formation of *Drosophila* olfactory avoidance can be inhibited by ubiquitous, post-training, inducible disruption of the circadian system. In this report, we limit intervention to the dorsal anterior lateral neurons, cells important for memory formation but not considered a part of the central clock. Post-training induction of “dominant negative” proteins or RNAi directed against either the *clock* or *cycle* genes in dorsal anterior lateral neurons disrupts 3-day memory. This experimental design minimizes indirect effects due to abnormal neuronal development, altered sensory processing at the time of training, effects of widespread ectopic expression of inhibiting proteins, and decreases the likelihood of “off-target” effects contributing to the disruption of memory. Induction prior to testing does not have any effect, likely ruling out an effect on retrieval. The transgene inductions mildly affect circadian locomotor rhythmicity and sleep but similarly to how these are affected in the parents of the progeny. Therefore, the effects on memory are very unlikely to be attributable to alterations in circadian locomotor activity or sleep. Paradoxically we also show that mutants in two of the circadian genes have normal 3-day memory. Thus, while global mutations in circadian genes do not impair memory formation, spatially and temporally restricted interventions in neurons do. We speculate that this discrepancy resembles a previously described developmental phenotype involving the central and a peripheral clock. In both contexts, systems-level compensation may allow events to occur independently of a functional circadian clock.

## Introduction

The identification of the core genes and molecules in the circadian clock stands as the greatest testimony to the insights and experimental strategy that Seymour Benzer and his laboratory initiated (Bonini, 2008). The subsequent demonstration of the phylogenetic conservation of these genes, the logic and the feedback molecular loops reinforces the far-sightedness of Benzer’s choice to use *Drosophila* as his organism and forward genetics as his experimental entry point into neurogenetics (Siwicki et al., 2018). Circadian rhythms are an “emergent” property of the ~240 neurons that constitute the central clock in the adult fly brain (Reinhard et al., 2024). These neurons are subdivided into different functional clusters each contributing specific “subfunctions” that integrate into, and comprise, circadian regulation of the rhythmic locomotor pattern exhibited across the daytime and nighttime. The different clusters are divided into the small and large lateral ventral neurons (sLNvs and l-LNvs), the lateral dorsal and posterior neurons (LNds and LPNs), and the dorsal DN1, DN2 and DN3 groups. Recent advances using single cell transcriptomic analyses, circuit mapping, synaptic connectome mapping and functional assays have further differentiated these clusters into subsets of cells and suggest at least 17 different functional subunits (Johard et al., 2009; Hermann et al., 2012; Hermann-Luibl et al., 2014; Schubert et al., 2017; Delventhal et al., 2019; Sekiguchi et al., 2020; Menagazzi et al., 2020; Shafer et al., 2022; Le et al., 2024; Reinhard et al., 2024; Ehrlich et al., 2024). For example, the 5th s-LNv is now thought to function more similarly to one of the LNd neurons and together they comprise a functional subunit (Johard et al., 2009; Hermann-Luibl et al., 2014; Schubert et al., 2017; Menagazzi et al., 2020; Sekiguchi et al., 2020; Shafer et al., 2022; Le et al., 2024). Continued experimental analyses will be needed to further understand the expression and functional distinctions of specific subgroups of cells within one cluster, and to understand how the clusters communicate with each other, their “hierarchical relationships” as well as downstream, output signaling from the clock.

Many of the physiological processes that are under circadian control are directly regulated by peripheral clocks (Hardin et al., 2003; Ito and Tomioka, 2016; Sehgal, 2016; Di Cara and King-Jones, 2016; Selcho et al., 2017; Mark et al., 2021; Wegener et al., 2024; Yildirim et al., 2022). Peripheral clocks take the central timekeeping information (based mostly on the light:dark cycle) and modify it to provide “time-of-day” peaks and valleys to different physiological processes in the relevant cells/tissues. In mammals, most if not all cells contain the full complement of circadian molecules. Thus, most cells and tissues are believed to be capable of functioning as peripheral clocks. In flies, the distribution of the suite of circadian molecules is more limited. Numerous peripheral clocks have been discovered, but their functional relationship to the central clock varies, ranging from total subservience to being semi-or completely autonomous.

Another major contribution of Benzer’s laboratory was pioneering the genetic approach to the study of learning and memory formation. The initial discoveries of cyclic adenosine monophosphate (cAMP) and its metabolism as core components of the “memory machine” paralleled work in *Aplysia* (Alberini, 1999). cAMP-responsive signaling and gene expression continue to be an active area of investigation, contributing new findings to synaptic plasticity, associativity, allocation of neurons into memory engrams, and memory formation (Lee, 2015; Rashid et al., 2016; Havekes and Abel, 2017; Huang et al., 2025). Fittingly, the intersection of these two important neurobiological processes (circadian biology and memory formation) is a newer frontier topic of research. Circadian rhythms have been shown to affect learning and memory formation in mammals and invertebrates, but the details and mechanisms remain unclear (Fernandez et al., 2003; Lyons et al., 2005; Lyons and Roman, 2008; Eckel-Mahan et al., 2008; Gerstner et al., 2009; Gerstner and Yin, 2010; Phan et al., 2011; Lubinski and Page, 2016; Xia and Storm, 2017; Price et al., 2016; Rawashdeh et al., 2018; Snider et al., 2018). In *Drosophila*, this dependency has been demonstrated using two different paradigms for long-term memory, the learned suppression of male courtship and olfactory avoidance (Sakai et al., 2004; Lyons and Roman, 2008; Gerstner et al., 2011; Fropf et al., 2014; Fropf et al., 2018; Inami et al., 2020; Inami et al., 2021; Inami et al., 2022; Yin et al., 2023). For both behaviors, the necessity of circadian molecules is clear but detailed molecular mechanisms are largely unknown. In courtship suppression, this requirement involves cells that are part of the central clock (Suzuki et al., 2022).

In this report, we show that some circadian genes are needed in the dorsal anterior lateral (DAL) neurons, a pair of cells previously shown to be required for memory of olfactory avoidance, but which are not thought to be part of the canonical central clock (Chen et al., 2012; Lin et al., 2021). Intriguingly, we show that (ubiquitous) loss-of-function mutations in some of the core clock genes do not seem to affect 3-day (3d) memory, while acute, inducible disruption of clock genes in only the DAL neurons does affect consolidation. We speculate that, in mutants, systems-level compensatory mechanisms within the central clock cells or its interactions with other neurons involved in the memory circuit may substitute for the timekeeping functions normally provided by an intact circadian clock.

## Materials and methods

### Flies

All the flies used have been validated and published previously. For the reporter experiments, the G0338-or G0431-Gal4 driver lines (which are both expressed in DAL neurons) are combined with the two other transgenes (*period-*promoter-FRT-*luciferase* and 20xUAS-FLP) that comprise the LABL system (Chen et al., 2012; Lin et al., 2021; Johnstone et al., 2022). For olfactory behavior, circadian and sleep experiments, progeny from a cross between one parent homozygous for two different transgenes and a second parent homozygous for a third transgene are used. The DAL specific driver line (G0431) is combined with the *tubulin*P-Gal80^ts^ transgene in the doubly transgenic parent (*w^1118^*; G0431-Gal4; *tubulin*P-GAL80^ts^). For the rest of this report, this genotype will be shortened to G0431; *tubulin*P-Gal80^ts^. The other parent is one of: [*w^1118^*; +/+; UAS-*clk^Δ^* (Tanoue et al., 2004)], [*w^1118^*; UAS-*cyc^Δ^*; +/+ (Tanoue et al., 2004)], [*w^1118^*; UAS-*clk^RNAi^*; +/+ (VDRC 107576)] or [*w^1118^*; UAS-*cyc*^RNAi^; +/+ (VDRC 110455)]. These four genotypes will be shortened to: UAS-*clk^Δ^*, UAS-*cyc^Δ^*, UAS-*clk*^RNAi^ and UAS-*cy*c^RNAi^. The *tubulin*P-Gal80^ts^ transgene makes the Gal80^ts^ protein ubiquitously. In the progeny flies that contain all three transgenes, at low temperature (20°C) the Gal80^ts^ protein is active and represses Gal4 from acting. At the restrictive temperature (29°C), the Gal80^ts^ protein is inactive and Gal4 becomes active. For assays of circadian locomotor activity and sleep, the parents and progeny from two of the crosses (those involving UAS-*clk*^Δ^ _or UAS-*cyc*^Δ^) are used. The UAS-*cyc^Δ^* _and UAS-*clk^Δ^* _flies are from P. Hardin, although the UAS-*clk*^Δ^ stock is available through BDSC (#36319). The *cyc^o^*, *tim^o^* stocks along with their isogenic wild-type control flies are from A. Sehgal. The G0338 and G0431-Gal4 driver lines and the doubly transgenic parent (G0431; *tubulin*P-GAL80^ts^) are from T. Tully.

### LABL reporter assay

Flies of the correct genotype (with the DAL-specific Gal4 driver lines [either G0338 or G0431], the LABL transgenic reporter and the UAS-FLP transgene) are assayed and analyzed as described previously (Johnstone et al., 2022).

### Olfactory avoidance behavior

Behavioral training is done as described previously with a few modifications to the induction conditions (Yin et al., 2023). Young flies (roughly 1 week of age) are used for behavior. Flies are collected and kept in groups of 100–150 individuals/vial and entrained to a 12-h lights on: 12-h lights off schedule at 20°C for 3d prior to training. Training starts between ZT = 14 and ZT = 16, and flies are returned to their dark period and kept at 20°C until testing. Testing always occurs around ZT = 16 to ZT = 18. For flies that undergo heat-shock induction, vials are shifted to incubators at 29°C under the same light:dark regimen.

Flies are trained in the olfactory avoidance-training paradigm developed by Tully and Quinn and modified to allow for automated training sessions (Tully and Quinn, 1985; Tully et al., 1994). A single-cycle of training consists of 90 s exposure to ambient air; 60 s of electric shock [the unconditioned stimulus which consists of 70 V pulses lasting 1.5 s and administered every 5 s (12 total)] accompanied by simultaneous exposure to 1 odor (the conditioned stimulus, CS+); 45 s of ambient air exposure to clear the first odor; 60 s of exposure to the second odor with no shock (the CS-condition), 45 s of ambient air to clear the second odor. This single training trial takes about 2.6 min. Spaced training consists of 10 single cycles separated by 15-min rest intervals. This training requires about 2 h 50 min of time. The odors that are used are 3-octanol and 4-methylcyclohexanol carried on an airstream.

Testing is done by placing flies in a choice point and giving them 2 min to decide between the CS + and CS-stimuli. The performance index = [the number of flies making the correct choice]−[the number of flies making the incorrect choice]/total number of flies, multiplied by 100. To avoid odor-avoidance biases, we calculate the performance index of every single N by taking an average performance of two groups of flies, one trained with 3-octanol as CS+, and the other with 4-methylcyclohexanol as the CS+. Flies were trained in a balanced manner, such that the sequence of shock-paired odors alternates, as well as the assignment of left vs. right arm at the choice point during testing. Data is presented as the standard error of the mean, and the Student’s T-test was used to evaluate statistical significance in pairwise comparisons.

### Circadian locomotor activity

Locomotor activity is measured as described previously using both parents and progeny from the behavioral crosses involving G0431; *tubulin*P-Gal80^ts^ and UAS-*clk*^Δ^ or UAS-*cyc*^Δ^ flies. Young flies (about 1-2-days post-eclosion) are entrained on a 12:12 light–dark cycle for 2 days before being loaded into the *Drosophila* Activity Monitor (DAM) System. The flies are allowed to recover for 2 days under the same light–dark cycle, at 20°C, shifted to constant darkness for 2 days, and then shifted to 29°C heat-shock until the end of the experiment. The SleepMat program is used to analyze periodicity and to generate the periodogram, and a custom program described previously is used to analyze and plot circadian locomotor activity (Sisobhan et al., 2022; Yin et al., 2023).

### Sleep

Sleep is measured as described previously using both parents and progeny from the behavioral crosses involving G0431; *tubulin*P-Gal80^ts^ and UAS-*clk*^Δ^ or UAS-*cyc*^Δ^ flies. Young flies (about 1-2-days post-eclosion) are entrained on a 12:12 light–dark cycle for 2 days at 20°C before being loaded into the DAM System. The flies are allowed to recover under the same light–dark cycle and temperature for 2 days, followed by a shift to 29°C starting at ZT 23 on day 3 until the end of the experiment. A custom program described previous is used to analyze and plot sleep figure (Yin et al., 2023).

## Results

### A *period*-promoter based reporter cycles in DAL neurons

Previously we showed that ubiquitous, post-training, *inducible* disruption of the circadian system interferes with 3-day (3d) memory formation (Yin et al., 2023). Chen et al. inferred that the circadian genes are expressed in the dorsal-anterior lateral (DAL) neurons, a pair of neurons located outside of the canonical central clock network, and showed that DAL neurons are important for 1d memory (Chen et al., 2012). Subsequent single-cell transcriptomic work shows that the circadian genes *cycle*, *pdp1* and *cry* are likely expressed in the DAL neurons (Crocker et al., 2016; Supplemental Table 6, #287, #676 and #712). In this report we ask whether the circadian genes in those neurons are important for 3d memory.

**Figure 1:**
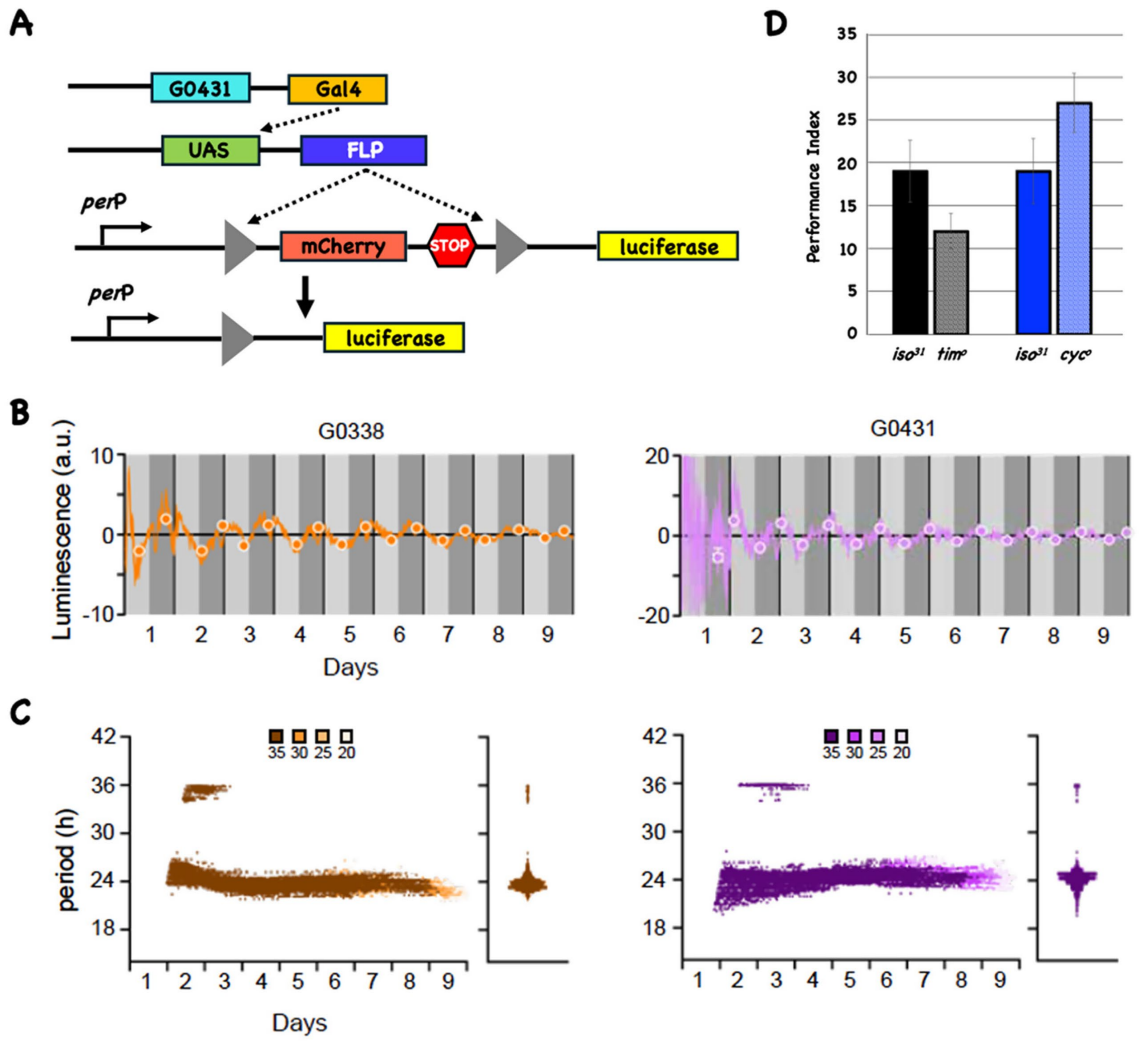
The LABL reporter oscillates in DAL neurons and circadian mutants have normal 3-day memory. **(A)** The LABL reporter system. Cartoon of three transgenes (shown as line cartoons) that are present in the same fly. The first transgene expresses the transcription factor Gal4 in a DAL-specific anatomical pattern. The Gal4 protein acts on UAS sequences to make the FLP recombinase (second line cartoon). The third transgene contains a fragment from the *period* promoter (*per*P) and a FLP-out cassette (triangles flanking mCherry, followed by stop codons in all reading frames) upstream of the coding region for luciferase (missing its ATG start codon). The black arrow signifies the start of transcription and translation. In the presence of FLP, recombination at the FRT sites results in deletion of one FRT site and the intervening sequences, including the translation termination codons (fourth line cartoon). **(B)** FLP expression in DAL neurons results in oscillating reporter activity. When the LABL system is driven using the DAL-specific Gal4 lines (G0338 [left plot in orange] or G0431 [right plot in magenta]), transcriptional activity oscillates. Luminescence (in arbitrary units) is plotted as a function of time. Daytime and nighttime periods are shown in light and dark gray and the vertical black lines indicate a 24-h period. The best fit maxima and minima for each 24-h period are shown with circles. **(C)** LABL oscillates with an approximate 24-h periodicity in DAL neurons. Data from the LABL experiments (*n* = 4) is analyzed as before (Johnstone et al., 2022). Morlet wave analysis for the entire 9 days shows that the periodicity of both reporters is about 24 h. Confidence intervals are shown in shades of color and indicated above the figure. The violin plots indicate the spread of all the data points. **(D)** Mutations in *tim* and *cyc* do not significantly affect 3d memory. The 3d performance indices for wild-type (*iso^31^*) and mutant stocks are plotted as a function of genotype. There is no significant difference in either mutant when compared to its wild type control.

Initially we use the LABL reporter system to ask if there is detectable oscillatory transcriptional activity in DAL neurons (Johnstone et al., 2022). Reporter activity is an indirect measure for CLK and CYC activity since the LABL reporter contains a minimal *period* promoter that results in oscillatory activity if CLK and CYC bind to a consensus E-box sequence (Bargiello et al., 1987). Figure 1A cartoons the necessary components in this system, and we test it in DAL using the G0338 or G0431 Gal4 driver lines (Chen et al., 2012).

Figure 1B shows reporter activity when FLP is expressed in DAL neurons. Luminescence (in arbitrary units) is plotted as a function of time for the G0338- (left side plot in orange) and G0431-driven (right side plot in purple) reporters, with the subjective day (light gray) and subjective night (dark gray) periods indicated. The closed circles represent the best fits for maxima and minima of the oscillating activity each day. Although the amplitudes are low, both G0338-and G0431-driven reporter activities oscillate. Morlet wavelet analysis (Figure 1C) indicates that the periodicity of the oscillations cluster around 24 h for all confidence intervals, consistent with circadian-regulated transcription. The violin plots to the right of each Morlet wavelet analysis show that the periodicities across the entire 9d interval cluster around 24 h for reporters activated using both drivers. Since CLK/CYC heterodimers are major contributors to oscillating transcription from the *period* promoter, they are likely to be expressed and active in DAL neurons, confirming findings from prior work (Bargiello et al., 1987; Chen et al., 2012; Crocker et al., 2016). Crocker et al. reported detecting the expression of the circadian genes *cyc*, *pdp1* and *cry* in DAL neurons using a scRNA sequencing approach (Crocker et al., 2016).

### Post-training, induced disruption of *clk* or *cyc* in DAL neurons disrupts 3d memory

**Figure 2:**
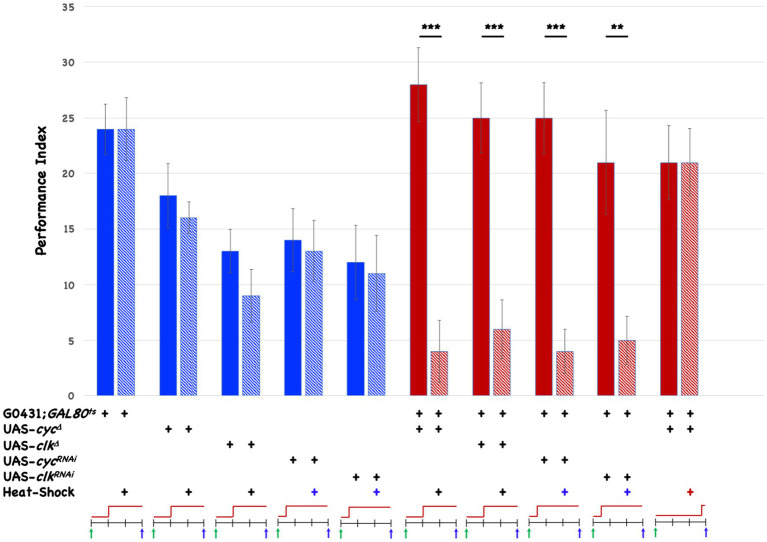
Post-training induction of genes that disrupt *cyc* and *clk* affects 3d memory. The Performance Index (PI) of flies is plotted as a function of genotype and treatment. The PIs of five different parental stocks are shown using blue bars, while the PIs of progeny from pairwise crosses are depicted with red bars. One parent in each cross is G0431; *tubulin*P-GAL80^ts^. The other parent in each cross is UAS-*cyc*^Δ^ (P. Hardin), UAS-*clk*^Δ^ (Bloomington #36319), UAS-*cyc*^RNAi^ (VDRC ^#^110455), or UAS-*clk*^RNAi^ (VDRC ^#^107576). The genotypes are indicated in the tabular part of the Figure. Heat-shock induction is shown with a +, and the resulting PIs are shown in striped-blue or striped-red bars, while PIs from uninduced flies are shown in solid-colored bars. The cartoon below the table indicates the timeline of the experiment. Each vertical line indicates a 24-h interval, and the training is indicated with a green arrow, while testing is shown with a blue arrow. The red lines show when heat-shock is delivered, and the row of the table with colored + signs indicate the three different times when heat-shock is started relative to the end of training (two experimental timepoints, and one control timepoint for the retrieval experiment). Error bars indicate SEM. Significant pairwise comparisons are indicated. ** *p* < 0.01, *** *p* < 0.001, T-test. *N* = 8 for all experiments. All other pairwise comparisons are not significantly different.

Since CLK and CYC are likely to be expressed in DAL neurons and to function together to contribute to rhythmic LABL activity (Figures 1B,C), we inducibly disrupt them in DAL neurons to ask about the possible requirement for circadian genes in memory formation. The G0431-Gal4 driver is combined with the ubiquitously expressed *tubulin*P-Gal80^ts^ transgene to test four different target UAS-transgenes in triply transgenic flies. At low temperature (20°C), the Gal80^ts^ protein binds Gal4 and prevents expression from the UAS transgene. At the non-permissive temperature (29°C), the Gal80^ts^ protein becomes inactive and Gal4 activates transcription from the UAS target transgene. The four different UAS-transgenes make truncated, “dominant negative” versions of CYC or CLK (CYC^Δ^ or CLK^Δ^), or RNAi directed against each respective gene. All parental stocks or triply transgenic (progeny) flies are trained with 10 cycles of spaced training, induced or not beginning 6–24 h after the end of training, and tested for 3d memory. Figure 2 shows the results when we test the effects of post-training, inducible disruption on 3d memory.

Although baseline performance varies among the different stocks, induction in any of the parental lines has no effect on 3d memory (compare the 4 leftmost pairs of blue and blue-striped bars). However, triply transgenic flies (red and red-striped bars) exhibit deficits in 3d memory upon induction. The magnitude of the impairment is nearly identical whether the induced product is a dominant negative CYC^Δ^ or CLK^Δ^ protein, or an RNAi molecule targeted against the *cyc* or *clk* gene. Induction of the CYC^Δ^ encoding transgene 3 h prior to behavioral testing (rightmost pair of bars) does not affect performance, showing that the CYC^Δ^ protein is not likely to affect retrieval. Taken collectively it is very likely that post-training disruption of *cyc* or *clk* in DAL neurons disrupts 3d memory.

### The *cyc^o^* or *tim^o^* mutants show normal 3d memory

Chen et al. previously showed that “classical” circadian mutants in *Drosophila* (other than *period*) do not affect 1-day (1d) memory of olfactory avoidance behavior (Chen et al., 2012). Sakai et al. reported identical results using the suppression of male courtship behavior testing at a later timepoint (Inami et al., 2021; Inami et al., 2022). Since we show inducible disruption of *cyc* or *clk* affects 3d memory (Figure 2), we test two widely studied mutants (*tim^o^* and *cyc^o^*) for their possible effects on 3d memory (see Figure 1D). The mutants and their isogenic wild-type control stocks were entrained to a 12:12 light:dark schedule for 3 days and then trained with 10 cycles of spaced training beginning about ZT16 under dim red lighting. Following training flies are returned to their pre-training light:dark cycle and tested for 3d memory. The solid bars represent the performance of the wild type controls, while the hatched bars show the results for the mutants. Under these conditions we do not detect any significant differences in 3d memory between mutants and wild-type flies.

### Inducible disruptions of *clk* or *cyc* modestly affect circadian locomotor activity and sleep but are probably not responsible for behavioral deficits

**Figure 3:**
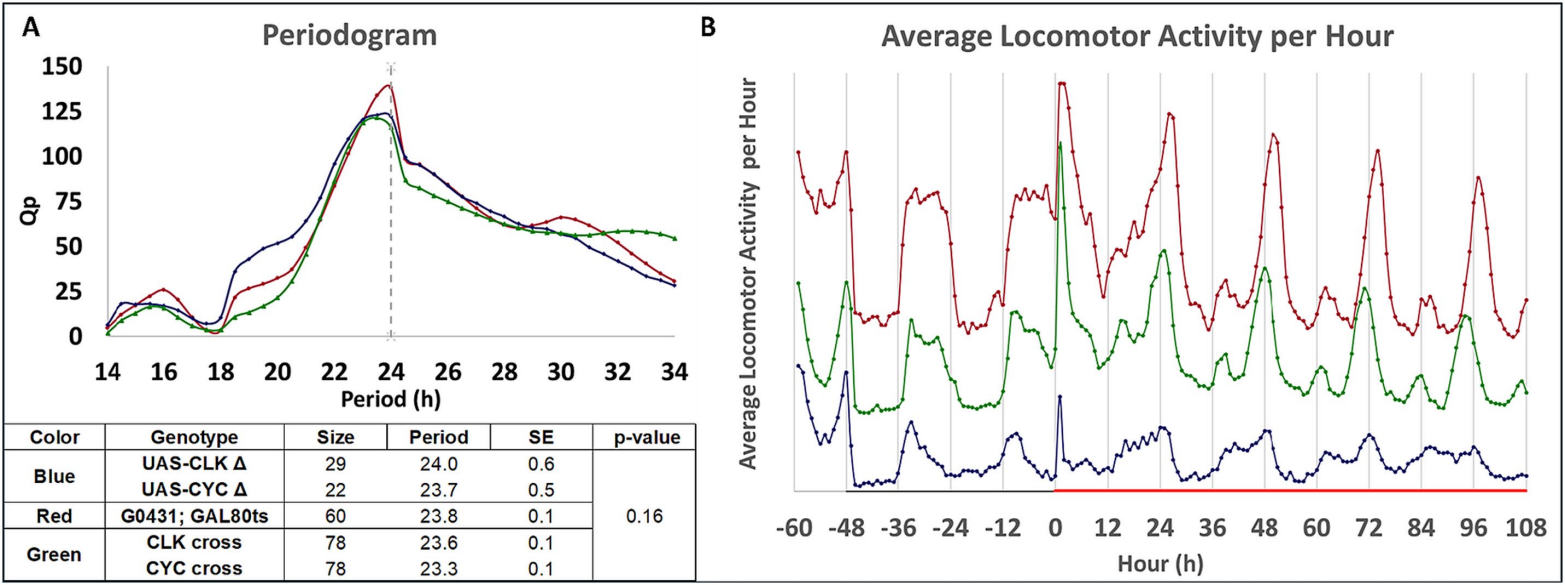
Effect of heat-shock induction on circadian locomotor activity. **(A)** Both parents and progeny exhibit mainly 24-h periodicity in locomotor activity after heat-shock. Male parents and progeny from two matings (G0431; *tubulin*P-GAL80^ts^ x UAS-*cyc*^Δ^ or G0431; *tubulin*P-GAL80^ts^ x UAS-*clk^Δ^*) were collected, placed in DAM tubes in a 20°C incubator, and entrained to light:dark cycles (12:12) for three days. The incubator is shifted to constant darkness for two days, then shifted to 29°C. Fly locomotor activity is monitored across the entire duration. The Periodogram data is based on the average number of beam breaks binned hourly across three days of time in constant darkness and at high temperature. The frequency of periodicity is plotted as a function of period length (in hours). Different colored lines represent the various genotypes: red for G0431; *tubulin*P-GAL80^ts^, blue for UAS-*cyc^Δ^* and UAS *clk^Δ^* (which are nearly identical and thus grouped together) and green for the progeny of both crosses (which were nearly identical and thus grouped together). Quantitation of the activity is presented in the Table below the Periodogram. The different genotypes and their color codes used in the Periodogram are indicated, as is the *N* = size, the calculated average periodicity, and the standard error of the mean for each group. Pairwise comparisons do not show any significant differences. **(B)** The architecture and relative levels of locomotor activity in the parents and progeny from the two crosses. The average locomotor activity is plotted as a function of experimental time, with 0 representing the onset of the shift from 20°C to 29°C. Negative and positive numbers indicate the time (in hours) relative to the 20°C to 29°C shift in temperature. The green trace (progeny from both crosses grouped together) is close to the average value for the two parents (red and blue traces).

Mechanistically, how is our inducible intervention affecting memory consolidation? To assess the possible effects of induction on circadian rhythmicity (Figure 3), we use the DAM to measure locomotor activity from the two different crosses ([G0431; *tubulin*P-GAL80^ts^ x UAS-*cyc^Δ^*] and [G0431; *tubulin*P-GAL80^ts^ x UAS-*clk^Δ^*]) that are used for behavior. Flies are entrained to a 12:12 light:dark cycle for two days, shifted to constant darkness for two days, and then maintained in darkness but shifted to 29°C to induce the heat-shock transgenes. Locomotor activity is recorded and analyzed over the following three full days. Rhythmicity is tested using a previously published method, and the resulting periodogram is presented in Figure 3A (Sisobhan et al., 2022). The quantitative analysis is shown in Tabular form at the bottom of Figure 3A. Five different groups are evaluated (the G0431; *tubulin*-Gal80^ts^ parent, the UAS-*cyc^Δ^* parent, the UAS-*clk^Δ^* parent, and the two progeny from the crosses). Because the two UAS parents behave identically, they are grouped together in the periodogram at the top of Figure 3A and represented using a blue trace. Similarly, the two progeny are nearly identical and grouped together and represented using a green trace. The G0431-Gal4; *tubulin*-Gal80^ts^ parent is represented using a red trace. The frequency of different periods is plotted as a function of period length. Induction does not substantially alter the periodicity of any of the groups including the progeny which are the only groups that disrupt memory upon induction.

Figure 3B shows the locomotor activity profiles for the same three groups (UAS parents, G0431; *tubulin*-Gal80^ts^ parent, and the progeny) plotted as a function of time across the experiment. The origin of the x-axis indicates when heat-shock induction (20°C to 29°C) begins and the negative and positive numbers denote the hours prior to, and after, induction. Locomotor activity of the progeny flies (green trace) appears as an arithmetic average of that of their parents (red and blue traces) and all flies maintain a similar general pattern of rhythmicity. In comparing the activity pre-and post-induction, both the parents (no memory deficits) and progeny (memory deficit) exhibit a subtle shift after induction (Figure 3B). They display a broadly distributed single peak during the subjective daytime period of constant darkness (−36 to −24 and −12 to 0 h prior to induction) and a much sharper and centered doublet activity peak post-induction (+24, +48). Since this change is not exclusive to the progeny of the crosses, this change is unlikely to contribute to the memory problems in those groups. ANOVA tests of the average periodicity for all genotypes also shows no significant statistical differences between groups, reinforcing the view that the subtle changes to circadian locomotor activity are unlikely to contribute to the memory deficits.

**Figure 4:**
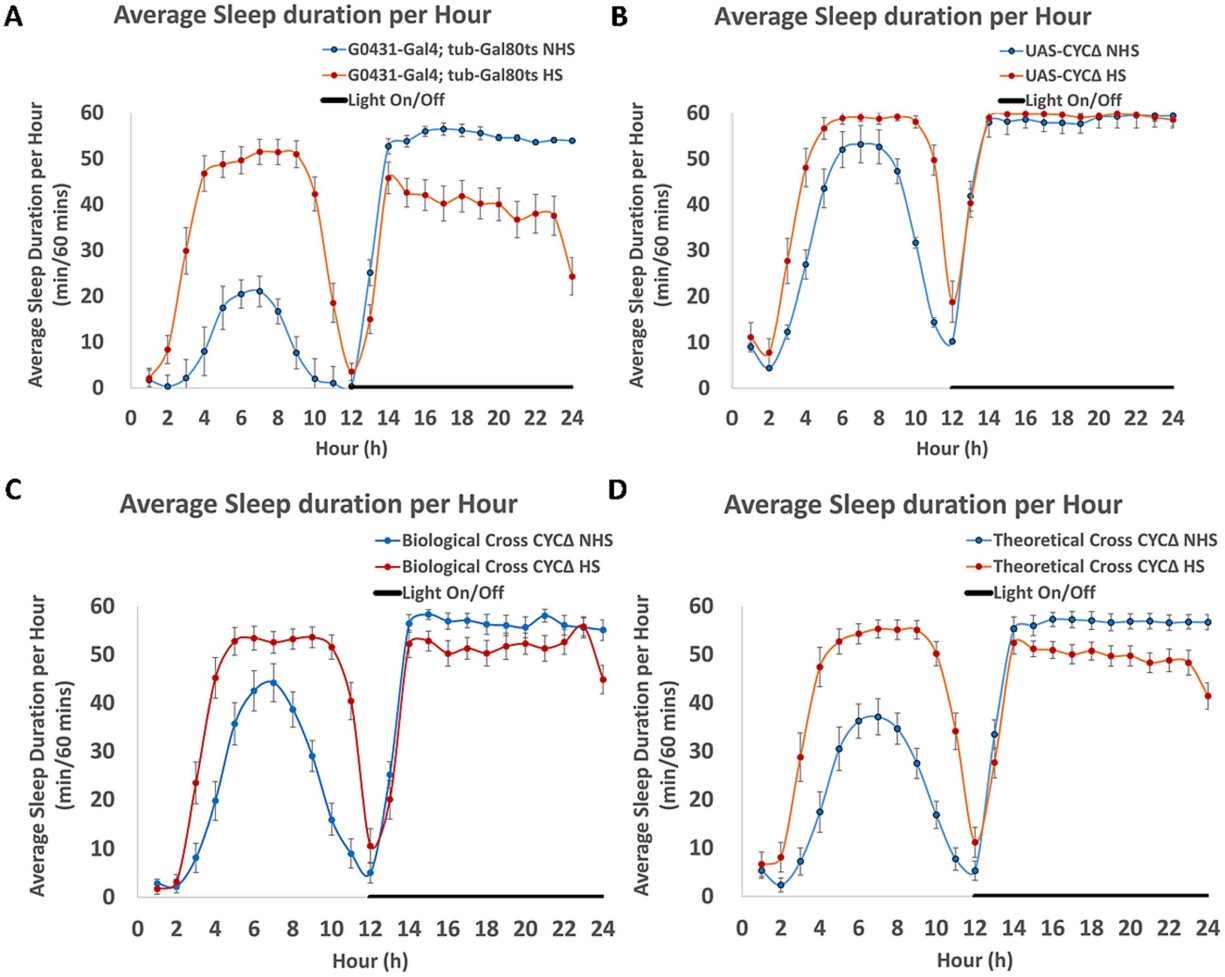
The sleep profile of parents and progeny exposed (or not) to heat-shock. **(A)** Male parents and progeny from a mating (G0431; *tubulin*P-GAL80^ts^ x UAS-*cyc*^Δ^) are collected, placed in DAM tubes in two separate 20°C incubators, and entrained to light:dark cycles (12:12) for three days. One incubator is shifted to high temperature (29°C) at ZT0, while the other remains at 20°C for multiple days. Locomotor activity is measured for the entire duration of the experiment, and sleep is calculated using a 5-min bin of inactivity as the criteria for sleep. The total amount of sleep is plotted as a function of time and genotype. The average duration of sleep (for 48-h prior to, and after induction) is shown for the two parents **(A,B)** and the progeny **(C)**. Flies that do not experience heat-shock are plotted in blue, while flies that undergo induction are shown in red. **D** shows the theoretical values if the actual sleep data from the parents **(A,B)** are averaged. For the G0431; tubulin-GAL80^ts^ parent, *N* = 54, for UAS-*cyc^Δ^*, *N* = 64, and for the progeny *N* = 64.

It is generally accepted that sleep is important for memory consolidation. Are our behavioral deficits in 3d memory attributable to disruptions to sleep? To investigate this, we use the DAM system to assess both steady-state and post-induction sleep profiles of the parents and progeny from the same crosses that are used for behavior. In Figure 4, we show the average sleep profiles across two days both prior to and after induction for the parents and progeny of one of the crosses. Panels A and B show the average sleep per hour for the G0431; *tubulin*-Gal80^ts^ and UAS-*cyc*^Δ^ parents. Panel C shows the sleep profile for the progeny from this cross. Analysis of the total sleep during the day and nighttime suggests that the values for the progeny are the average of the values for the two parents regardless of whether the flies are induced or not. This averaging is most apparent when the “theoretical” (arithmetic average, Panel D) of the parents is compared to the actual data of the biological cross (the progeny, Panel C). The same result is true for the other cross as well (see Supplementary Figure 1). The detailed analyses of sleep for all five groups are presented in Supplementary Figure 2. We also show the kinetics of the effects of induction on sleep levels and on the architecture of sleep in the progeny from both crosses (grouped together, see Supplementary Figure 3). In general, heat-shock induction increases the amount of daytime sleep and the average length of daytime bouts, which are consistent with a more consolidated sleep state. Part of this effect is attributable to heat-shock itself increasing sleep (Lenz et al., 2015). However, the average number of daytime bouts also increases, and this pattern is usually associated with poorer quality of sleep. The effects of heat-shock on nighttime sleep all trend in the direction of less sleep consolidation—decreases in total amounts, with increases in bout number and decreases in bout lengths. However, like the effects of induction on circadian rhythms, the changes in sleep in the behaviorally relevant (progeny) flies are very close to an arithmetic average of the effects of induction on the parents. The presumed induced protein(s) do not appear to appreciably alter either circadian locomotor activity or sleep patterns more than what heat-shock induction itself does in the parents. Thus, it seems quite unlikely that any of the circadian locomotor-activity or sleep-related changes are contributing to the behavioral deficits.

## Discussion

In this report we show that post-training disruption of *clk* or *cyc* using the G0431 driver disrupts 3d memory. Since the G0431 driver is quite specific (but not exclusive) to DAL neurons, we cannot positively state that our disruptions only occur there (Chen et al., 2012). However, it is very unlikely that G0431 drives substantial gene expression in central clock neurons or in other major neuronal clusters. Behaviorally, our interventions are limited to a time window during consolidation, beginning 6-24 h after the end of training. The timing of induction ensures that our manipulations do not affect processing of sensory information during training or short-term memory formation. Since induction of the uncrossed parents (used to derive the multi-transgenic progeny that we use) do not have any effects on memory formation, disruption requires the presence of all 3 transgenes that reside in the progeny of each cross and is not an effect of the inducing treatment alone (heat-shock). We see identical effects whether we express “dominant-negative” forms of CYC or CLK or use RNAi to “knock-down” either of the genes. Thus, it is unlikely that our results are due to “off-target” effects of the disrupting molecules, nor to overexpression of either RNA or protein itself. Acute induction immediately before testing does not affect 3d memory, suggesting that the disruptions are affecting consolidation itself and not retrieval. Taken collectively, these experiments suggest an important role for circadian genes in DAL neurons to support 3d memory. We suggest that CYC and CLK are likely to be present in DAL neurons because of oscillatory E-box activity measured using the LABL system. However, we do not directly show that our transgenic manipulations affect LABL activity in DAL neurons.

Classic mutations in *tim* or *cyc* do not affect 3d memory. How can a global disruption of circadian molecules have less effect than a more anatomically limited one? We showed previously that post-training, ubiquitously induced *vri* or *clk^jrk^* disrupt 3d memory (Yin et al., 2023). We hypothesize that some type of systems compensation can occur in mutants to allow memory formation to occur “normally.” When the circadian system is inducibly challenged we hypothesize that there is not sufficient time from the onset of the effects of induction to the first critical window when circadian rhythmicity is needed for memory consolidation. This brevity prevents compensatory mechanisms from intervening. In this report we further limit our induction to DAL neurons.

Circadian mutants (*per^o^*, *tim^o^*, *clk*, *cyc^o^*) have minimal effect on development, but local knock downs in the prothoracic gland (PG) are lethal (Di Cara and King-Jones, 2016). The PG is a “peripheral clock” which receives multiple signals (PTTH, insulin-like peptides, transforming growth factor *β*/activin, hedgehog, JEB/ALK and PVF) from different cells during development (Di Cara and King-Jones, 2016; Kannangara et al., 2020; Pan and O’Connor, 2021; Cavieres-Lepe et al., 2024). The PG integrates these signals and synthesizes the steroid hormone ecdysone which is required for timing larval molts and eclosion of flies from pupal cases. The synthesis and timed secretion of ecdysone is under circadian control and integration of the different input information occurs in the PG. A plausible “systems-level” hypothesis is that desynchronization of the central and peripheral clocks is detrimental, but lack of circadian molecules everywhere can be overcome. This result resembles recent work in mice where removing circadian molecules in a peripheral tissue results in desynchronization and toxicity, but loss of clock molecules in both central and peripheral clocks is not (Woodie et al., 2024).

We speculate that the DAL neurons function similarly in the adult brain, serving as a “brain-specific peripheral clock” for synchronizing circadian and behavior-specific events. We do not provide any direct evidence that DAL neurons are outside of the cell clusters in the canonical central clock. Chen et al. provided GRASP evidence that DAL synapses with neurons in the *α*/β lobes of the mushroom body. However, recent work under review suggests that the sLNv can synapse with mushroom body neurons as well, suggesting that core clock neurons can connect with those neurons (Ehrlich et al., under review). Future detailed experiments will be needed to further demonstrate that DAL neurons are independent from the central clock cells. Regardless of the final resolution to this issue, we hypothesize that DAL functions like a peripheral clock. It is now apparent that there is a significant amount of “plasticity” built into the circadian system, and this plasticity could be partly responsible for compensation (Schlichting et al., 2019a; Schlichting et al., 2019b). For example, different ratios of light and dark across the 24 h period can alter the hierarchy of cells in the central clock resulting in behavioral plasticity. Since light is the strongest zeitgeber in many organisms including insects, it is plausible that our entrainment of flies prior to behavioral training and our maintaining them on LD cycles after training is important for compensation. These experimental conditions could provide “timekeeping” information even in the absence of certain clock molecules (for example in mutants). Intriguingly mutants in *period* disrupt memory formation of both olfactory avoidance and courtship suppression, contrasting sharply with the lack of effect of all the other classic circadian mutants (*tim*, *clk, cyc*) that have been tested (Sakai et al., 2004; Chen et al., 2012). This exception hints strongly that the PER protein is necessary for compensation.

Our analyses show that inducibly disrupting *cyc* or *clk* in DAL has modest effects on circadian locomotor activity, and some effects on sleep. The architecture of locomotor activity across the day/night is mildly affected in its timing of peak activity but retains a rhythmicity of about 24 h. The effects on sleep in the progeny of crosses are mixed, since daytime sleep is increased but nighttime sleep is decreased. For both circadian rhythms and sleep, the effects of induction on the progeny are arithmetic sums of the effects of induction on the parents. Since only the progeny show induction-dependent effects on memory, it is most likely that the induction-dependent effects on circadian locomotor rhythmicity and sleep are not responsible for the deficits in memory.

If disrupting the clock molecules in DAL do not affect circadian locomotor rhythms and sleep, how do we think the memory deficits occur? We hypothesize that the circadian genes regulate the timing of some important aspects of memory consolidation. Synaptic connectivity and/or peptidergic signaling between DAL and the mushroom body neurons (where the “memory information” is likely processed and stored) could facilitate “time-of-day” information, time stamping, and/or oscillating bursts of protein synthesis (Chen et al., 2012; Noya et al., 2019; Bruning et al., 2019; Ehrlich et al., under review). Further experimentation will be needed to clarify these roles but our results suggest that circadian molecules contribute to the timing of important events required during memory formation and its consolidation and that this occurs outside of the central clock.

## Data Availability

The raw data supporting the conclusions of this article will be made available by the authors, without undue reservation.

## References

[ref1] Alberini C. M. (1999). Genes to remember. J. Exp. Biol. 202, 2887–2891. doi: 10.1242/jeb.202.21.2887, PMID: 10518471

[ref2] Bargiello T. T. Jackson F. R. Young M. W. (1987). Restoration of circadian behavioral rhythms by gene transfer in Drosophila. Nature 312, 752–754. doi: 10.1038/312752a0 6440029

[ref3] Bonini N. (2008). A tribute to Seymour Benzer, 1921-2007. Genetics 180, 1265–1273. doi: 10.1534/genetics.104.97782, PMID: 19001297 PMC2581933

[ref4] Bruning F. Noya S. B. Bange T. Koutsouli S. Rudolph J. D. Tyagarajan S. K.. (2019). Sleep-wake cycles drive daily dynamics of synaptic phosphorylation. Science 366:eaav3617. doi: 10.1126/science.aav3617, PMID: 31601740

[ref5] Cavieres-Lepe J. Amini E. Zabel M. Nassl D. R. Stanewsky R. Wegener C.. (2024). Timed receptor tyrosine kinase signaling couples the central and a peripheral circadian clock in Drosophila. Proc. Natl. Acad. Sci. USA 121:e2308067121. doi: 10.1073/pnas.2308067121 38442160 PMC10945756

[ref6] Chen C. C. Wu J. K. Lin H. Pai T. Fu T. E. Wu C. L.. (2012). Visualizing long-term memory formation in two neurons of the Drosophila brain. Science 335, 678–685. doi: 10.1126/science.1212735 22323813

[ref7] Crocker A. Guan X.-J. Murphy C. T. Murthy M. (2016). Cell-type-specific transcriptome analysis in the Drosophila mushroom body reveals memory-related changes in gene expression. Cell Rep. 15, 1580–1596. doi: 10.1016/j.celrep.2016.04.046, PMID: 27160913 PMC5047377

[ref8] Delventhal R. O’Connor R. M. Pantalia M. M. Ulgherait M. Kim H. X. Basturk M. K.. (2019). Dissection of central clock function in Drosophila through cell-specific CRISPR-mediated clock gene disruption. eLife 8:e48308. doi: 10.7554/eLife.48308 31613218 PMC6794090

[ref9] Di Cara F. King-Jones K. (2016). The circadian clock is a key driver of steroid hormone production in Drosophila. Curr. Biol. 26, 2469–2477. doi: 10.1016/j.cub.2016.07.004, PMID: 27546572

[ref11] Eckel-Mahan K. L. Phan T. Han S. Wang H. Chan G. C. Scheiner Z. S.. (2008). Circadian oscillation of hippocampal MAPK activity and cAMP: implications for memory persistence. Nat. Neurosci. 11, 1074–1082. doi: 10.1038/nn.2174, PMID: 19160506 PMC2772165

[ref12] Ehrlich A. Xu A. A. Luminari S. Kidd S. Treiber C. D. Russo J.. (2024). Tango-seq: overlaying transcriptomics on connectomics to identify neurons downstream of Drosophila clock neurons. Bio Rxiv. doi: 10.1101/2024.05.22.595372

[ref13] Fernandez R. I. Lyons L. C. Levenson J. Khabour O. Eskin A. (2003). Circadian modulation of long-term sensitization in Aplysia. Proc. Natl. Acad. Sci. USA 100, 14415–14420. doi: 10.1073/pnas.2336172100, PMID: 14610272 PMC283606

[ref14] Fropf R. Zhang J. Tanenhaus A. K. Fropf W. J. Siefkes E. Yin J. C. (2014). Time of day influences memory formation and dCREB2 proteins in Drosophila. Front. Syst. Neurosci. 8, 8–43. doi: 10.3389/fnsys.2014.00043, PMID: 24744705 PMC3978337

[ref15] Fropf R. Zhou H. Yin J. C. P. (2018). The clock gene period differentially regulates sleep and memory in Drosophila. Neurobiol. Learn. Mem. 153, 2–12. doi: 10.1016/j.nlm.2018.02.016, PMID: 29474956 PMC6064670

[ref16] Gerstner J. R. Lyons L. C. Wright K. P. Loh D. H. Rawashdeh O. Eckel-Mahan K. L.. (2009). Cycling behavior and memory formation. J. Neurosci. 29, 12824–12830. doi: 10.1523/JNEUROSCI.3353-09.2009, PMID: 19828795 PMC4077269

[ref17] Gerstner J. R. Vanderheyden W. M. Shaw P. J. Landry C. F. Yin J. C. (2011). Fatty-acid binding proteins modulate sleep and enhance long-term memory consolidation in Drosophila. PLoS One 6:e15890. doi: 10.1371/journal.pone.0015890, PMID: 21298037 PMC3029266

[ref18] Gerstner J. R. Yin J. C. (2010). Circadian rhythms and memory formation. Nat. Rev. Neurosci. 11, 577–588. doi: 10.1038/nrn2881, PMID: 20648063 PMC6544049

[ref19] Hardin P. E. Krishnan B. Houl J. H. Zheng H. Ng F. S. Dryer S. E.. (2003). Central and peripheral circadian oscillators in Drosophila. Novartis Found. Symp. 253, 140–150. doi: 10.1002/0470090839.ch11 14712919

[ref20] Havekes R. Abel T. (2017). The tired hippocampus: the molecular impact of sleep deprivation on hippocampal function. Curr. Opin. Neurobiol. 44, 13–19. doi: 10.1016/j.conb.2017.02.005, PMID: 28242433 PMC5511071

[ref21] Hermann C. Yoshii T. Dusik V. Helfrich-Forster C. (2012). Neuropeptide F Immunoreactive clock neurons modify evening locomotor activity and free-running period in *Drosophila melanogaster*. J. Comp. Neurol. 520, 970–987. doi: 10.1002/cne.22742, PMID: 21826659

[ref22] Hermann-Luibl C. Yoshii T. Senthilan P. R. Dircksen H. Helfrich-Forster C. (2014). The ion transport peptide is a new functional clock neuropeptide in the fruit fly *Drosophila melanogaster*. J. Neurosci. 34, 9522–9536. doi: 10.1523/JNEUROSCI.0111-14.2014, PMID: 25031396 PMC6608324

[ref23] Huang S. Piao C. Zhao Z. Beuschel C. B. Turrel O. Toppe D.. (2025). Enhanced memory despite severe sleep loss in Drosophila insomniac mutants. PLoS Biol. 23:e3003076. doi: 10.1371/journal.pbio.3003076, PMID: 40111981 PMC12135902

[ref24] Inami S. Sato S. Kondo S. Tanimoto H. Kitamoto T. Sakai T. (2020). Environmental light is required for maintenance of long-term memory in Drosophila. J. Neurosci. 40, 1427–1439. doi: 10.1523/JNEUROSCI.1282-19.2019, PMID: 31932417 PMC7044726

[ref25] Inami S. Sato T. Kurata Y. Suzuki Y. Kitamoto T. Sakai T. (2021). Consolidation and maintenance of long-term memory involve dual functions of the developmental regulator Apterous in clock neurons and mushroom bodies in the Drosophila brain. PLoS Biol. 19:e3001459. doi: 10.1371/journal.pbio.3001459, PMID: 34860826 PMC8641882

[ref26] Inami S. Sato T. Sakai T. (2022). Circadian neuropeptide-expressing clock neurons as regulators of long-term memory: molecular and cellular perspectives. Front. Mol. Neurosci. 15:934222. doi: 10.3389/fnmol.2022.934222, PMID: 35909447 PMC9326319

[ref27] Ito C. Tomioka K. (2016). Heterogeneity of the peripheral circadian systems in *Drosophila melanogaster*: a review. Front. Physiol. 7:8. doi: 10.3389/fphys.2016.00008, PMID: 26858652 PMC4731491

[ref28] Johard H. A. D. Yoishii T. Dircksen H. Cusumano P. Rouyer F. Helfrich-Forster C.. (2009). Peptidergic clock neurons in Drosophila: ion transport peptide and short neuropeptide F in subsets of dorsal and ventral lateral neurons. J. Comp. Neurol. 516, 59–73. doi: 10.1002/cne.22099, PMID: 19565664

[ref29] Johnstone P. S. Ogueta M. Akay O. Top I. Syed S. Stanewsky R.. (2022). Real time, in vivo measurement of neuronal and peripheral clocks in *Drosophila melanogaster*. eLife 11:e77029. doi: 10.7554/eLife.77029, PMID: 36190119 PMC9662830

[ref30] Kannangara J. R. Henstridge M. A. Parsons L. M. Kondo S. Mirth C. K. Warr C. G. (2020). A new role for neuropeptide F signaling in controlling developmental timing and body size in *Drosophila melanogaster*. Genetics 216, 135–144. doi: 10.1534/genetics.120.303475, PMID: 32675276 PMC7463290

[ref31] Le J. Q. Ma D. Dai X. Rosbash M. (2024). Light and dopamine impact two circadian neurons to promote morning wakefulness. Curr. Biol. 34, 3941–3954.e4. doi: 10.1016/j.cub.2024.07.056, PMID: 39142287 PMC11404089

[ref32] Lee D. (2015). Global and local missions of cAMP signaling in neuronal plasticity, learning, and memory. Front. Pharmacol. 4:161. doi: 10.3389/fphar.2015.00161 PMC4523784 26300775

[ref33] Lenz O. Xiong J. Nelson M. D. Raizen D. M. Williams J. A. (2015). FMRFamide signaling promotes stress-induced sleep in Drosophila. Brain Behav. Immun. 47, 141–148. doi: 10.1016/j.bbi.2014.12.028, PMID: 25668617 PMC4467992

[ref34] Lin H.-W. Chen C.-C. de Belle J. S. Tully T. Chiang A.-S. (2021). CREBA and CREBB in two identified neurons gate long-term memory formation in Drosophila. Proc. Natl. Acad. Sci. USA 118:e2100624118. doi: 10.1073/pnas.2100624118, PMID: 34507985 PMC8449312

[ref35] Lubinski A. J. Page T. L. (2016). The optic lobes regulate circadian rhythms of olfactory learning and memory in the cockroach. J. Biol. Rhythm. 31, 161–169. doi: 10.1177/0748730415622710, PMID: 26714872

[ref36] Lyons L. C. Rawashdeh O. Katzoff A. Susswein A. J. Eskin A. (2005). Circadian modulation of complex learning in diurnal and nocturnal Aplysia. Proc. Natl. Acad. Sci. USA 102, 12589–12594. doi: 10.1073/pnas.0503847102, PMID: 16116090 PMC1194922

[ref37] Lyons L. C. Roman G. (2008). Circadian modulation of short-term memory in Drosophila. Learn. Mem. 16, 19–27. doi: 10.1101/lm.1146009, PMID: 19117913 PMC2632854

[ref38] Mark B. Bustos-Gonzalez L. Cascallares G. Conejera F. Ewer J. (2021). The circadian clock gates Drosophila adult emergence by controlling the time course of metamorphosis. Proc. Natl. Acad. Sci. USA 118:e2023249118. doi: 10.1073/pnas.2023249118, PMID: 34183412 PMC8271606

[ref39] Menagazzi P. Beer K. Grebler V. Schlichting M. Schubert F. K. Helfrich-Forster C. (2020). A functional clock within the main morning and evening neurons of *D. melanogaster* is not sufficient for wild-type locomotor activity under changing day length. Front. Physiol. 11:229. doi: 10.3389/fphys.2020.00229 32273848 PMC7113387

[ref40] Noya S. B. Colameo D. Bruning F. Spinnler A. Mircsof D. Opitz L.. (2019). The forebrain synaptic transcriptome is organized by clocks but its proteome is driven by sleep. Science 366:eaav 2642. doi: 10.1126/science.aav2642, PMID: 31601739

[ref41] Pan X. O’Connor M. B. (2021). Coordination among multiple receptor tyrosine kinase signals controls Drosophila developmental timing and body size. Cell Rep. 36:109644. doi: 10.1016/j.celrep.2021.109644 34469735 PMC8428980

[ref42] Phan T. X. Chan G. C. Sindreu C. B. Eckel-Mahan K. L. Storm D. R. (2011). The diurnal oscillation of MAP (mitogen-activated protein) kinase and adenylyl cyclase activities in the hippocampus depends on the suprachiasmatic nucleus. J. Neurosci. 31, 10640–10647. doi: 10.1523/JNEUROSCI.6535-10.2011, PMID: 21775607 PMC3146036

[ref43] Price K. H. Dziema H. Aten S. Loeser J. Norona F. E. Hoyt K.. (2016). Modulation of learning and memory by the targeted deletion of the circadian clock gene Bmal1 in forebrain circuits. Behav. Brain Res. 308, 222–235. doi: 10.1016/j.bbr.2016.04.027, PMID: 27091299 PMC5344043

[ref44] Rashid A. J. Yan C. Mercaldo V. Hsiang H.-L. Park S. Cole C. J.. (2016). Competition between engrams influences fear memory formation and recall. Science 353, 383–387. doi: 10.1126/science.aaf0594, PMID: 27463673 PMC6737336

[ref45] Rawashdeh O. Parsons R. Maronde E. (2018). Clocking in time to gate memory processes: the circadian clock is part of the ins and outs of memory. Neural Plast. 2018:6238989. doi: 10.1155/2018/6238989, PMID: 29849561 PMC5925033

[ref46] Reinhard N. Fukuda A. Manoli G. Derksen E. Saito A. Moller G.. (2024). Synaptic connectome of the Drosophila circadian clock. Nat. Commun. 15:10392. doi: 10.1038/s41467-024-54694-0, PMID: 39638801 PMC11621569

[ref47] Sakai T. Tamura T. Kitamoto T. Kidokoro Y. (2004). A clock gene, period, plays a key role in long-term memory formation in Drosophila. Proc. Natl. Acad. Sci. USA 101, 16058–16063. doi: 10.1073/pnas.0401472101, PMID: 15522971 PMC528738

[ref48] Schlichting M. Diaz M. M. Xin J. Rosbash M. (2019a). Neuron-specific knockouts indicate the importance of network communication to Drosophila rhythmicity. eLife 8:e48301. doi: 10.7554/eLife.48301, PMID: 31613223 PMC6794074

[ref49] Schlichting M. Weidner P. Diaz M. Menegazzi P. Dalla Benetta E. Helfrich-Forster C.. (2019b). Light-mediated circuit switching in the Drosophila neuronal clock network. Curr. Biol. 29, 3266–3276.e3. doi: 10.1016/j.cub.2019.08.033, PMID: 31564496

[ref50] Schubert F. K. Hagedorn N. Yoshii T. Helfrich-Forster C. Rieger D. (2017). Neuroanatomical details of the lateral neurons of *Drosophila melanogaster* support their functional role in the circadian system. J. Comp. Neurol. 526, 1209–1231. doi: 10.1002/cne.24406 PMC5873451 29424420

[ref51] Sehgal A. (2016). “Control of metabolism by central and peripheral clocks in Drosophila” in A time for metabolism and hormones. eds. Sassone-Corsi P. Christen Y. Oxenham A. J. Popper A. N. (Cham: Springer). 28892334

[ref52] Sekiguchi M. Inoue K. Yang T. Luo D.-G. Yoshii T. (2020). A catalog of GAL4 drivers for labeling and manipulating circadian clock neurons in *Drosophila melanogaster*. J. Biol. Rhythm. 35, 207–213. doi: 10.1177/0748730419895154, PMID: 31856635

[ref53] Selcho M. Millan C. Palacios-Munoz A. Ruf F. Ubillo L. Chen J.. (2017). Central and peripheral clocks are coupled by a neuropeptide pathway in Drosophila. Nat. Commun. 8:15563. doi: 10.1038/ncomms15563, PMID: 28555616 PMC5459987

[ref54] Shafer O. T. Gutierrez G. J. Li K. Mildenhall A. Spira D. Marty J.. (2022). Connectomic analysis of the Drosophila lateral neuron clock cells reveals the synaptic basis of functional pacemaker classes. eLife 11:e79139. doi: 10.7554/eLife.79139, PMID: 35766361 PMC9365390

[ref55] Sisobhan S. Rosensweig C. Lear B. C. Allada R. (2022). Sleepmat: a new behavioral software program for sleep and circadian rhythms. Sleep 45:zsac195. doi: 10.1093/sleep/zsac195 35998317 PMC9742897

[ref56] Siwicki K. K. Hardin P. E. Price J. L. (2018). Reflections on contributing to "big discoveries" about the fly clock: our fortunate paths as post-docs with 2017 Nobel laureates Jeff hall, Michael Rosbash, and Mike Young. Neurobiol. Sleep Circadian Rhythms 5:58067. doi: 10.1016/j.nbscr.2018.02.004 PMC6584674 31236512

[ref57] Snider K. H. Sullivan K. A. Obrietan K. (2018). Circadian regulation of hippocampal-dependent memory: circuits, synapses and molecular mechanisms. Neural Plast. 2018:7292540. doi: 10.1155/2018/729254 29593785 PMC5822921

[ref58] Suzuki Y. Kurata Y. Sakai T. (2022). Dorsal-lateral clock neurons modulate consolidation and maintenance of long-term memory in Drosophila. Genes Cells 27, 266–279. doi: 10.1111/gtc.12923, PMID: 35094465

[ref59] Tanoue S. Krishnan P. Dryer S. E. Hardin P. E. (2004). Circadian clocks in antennal neurons are necessary and sufficient for olfaction rhythms in Drosophila. Curr. Biol. 14, 638–649. doi: 10.1016/j.cub.2004.04.009, PMID: 15084278

[ref9001] Tully T. Quinn W. G. (1985). Classical conditioning and retention in normal and mutant Drosophila melanogaster. J. Comp. Physiol. A. 157, 263–277. doi: 10.1007/BF01350033 3939242

[ref9002] Tully T. Preat T. Boynton S. C. Del Vecchio M. (1994). Genetic dissection of consolidated memory in Drosophila. Cell. 79, 35–47. doi: 10.1016/0092-8674(94)90398-0 7923375

[ref60] Wegener C. Amini E. Cavieres-Lepe J. Ewer J. (2024). Neuronal and endocrine mechanisms underlying the circadian gating of eclosion: insights from Drosophila. Curr Opin Insect Sci. 66:101286. doi: 10.1016/j.cois.2024.101286, PMID: 39461671

[ref61] Woodie L. N. Alberto A. J. Krusen B. M. Melink L. C. Lazar M. A. (2024). Genetic synchronization of the brain and liver molecular clocks defend against chrono-metabolic disease. Proc. Natl. Acad. Sci. USA 121:e2417678121. doi: 10.1073/pnas.2417678121, PMID: 39665757 PMC11665897

[ref62] Xia Z. Storm D. (2017). Role of circadian rhythm and REM sleep for memory consolidation. Neurosci. Res. 118, 13–20. doi: 10.1016/j.neures.2017.04.011, PMID: 28434990 PMC8051942

[ref63] Yildirim E. Curtis R. Hwangbo D.-S. (2022). Roles of peripheral clocks: lessons from the fly. FEBS Lett. 596, 263–293. doi: 10.1002/1873-3468.14251, PMID: 34862983 PMC8844272

[ref64] Yin J. C. P. Cui E. Hardin P. E. Zhou H. (2023). Circadian disruption of memory consolidation in Drosophila. Front. Syst. Neurosci. 17:1129152. doi: 10.3389/fnsys.2023.1129152, PMID: 37034015 PMC10073699

