# Correction: The circadian genes are required in DAL neurons for *Drosophila* long-term memory formation

**DOI:** 10.3389/fnins.2025.1683967

**Published:** 2025-09-03

**Authors:** Jerry C. P. Yin, Ethan Cui, Peter S. Johnstone, Deniz Top, Grace Boekhoff-Falk, Hong Zhou

**Affiliations:** ^1^Laboratory of Genetics, School of Medicine and Public Health, University of Wisconsin-Madison, Madison, WI, United States; ^2^Neurology Department, School of Medicine and Public Health, University of Wisconsin-Madison, Madison, WI, United States; ^3^Department of Cell Biology, University of Alberta, Edmonton, AB, Canada; ^4^Department of Cell and Regenerative Biology, School of Medicine and Public Health, University of Wisconsin-Madison, Madison, WI, United States

**Keywords:** memory formation, circadian, *Drosophila*, DAL neurons, peripheral clock, mutants

There was a mistake in Figure 1 and the caption of Figure 1 as published. The stock labeled G0388 should be G0338. The corrected Figure 1 and its caption appear below.

**Figure 1:**
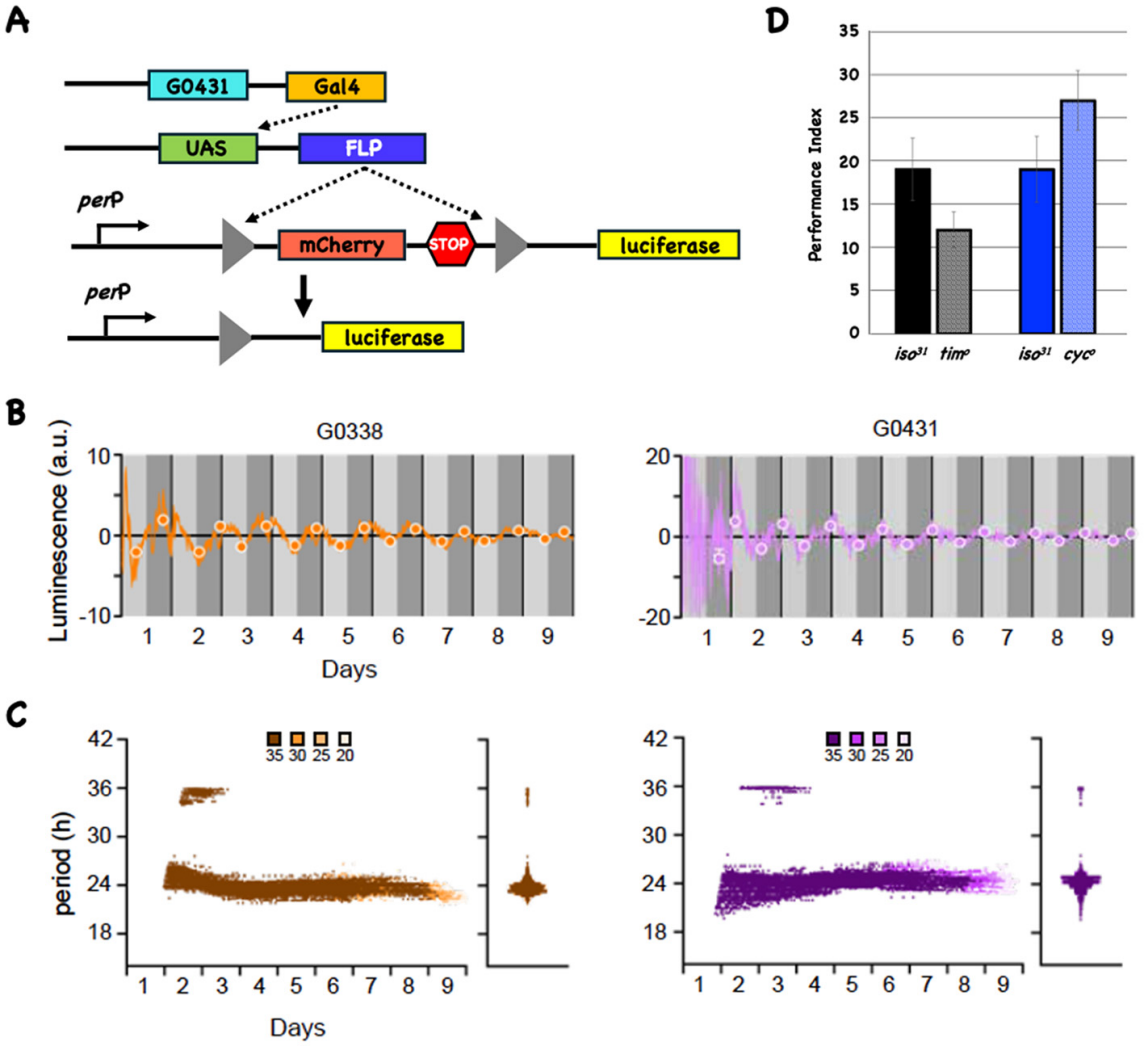
The LABL reporter oscillates in DAL neurons and circadian mutants have normal 3-day memory. **(A)** The LABL reporter system. Cartoon of three transgenes (shown as line cartoons) that are present in the same fly. The first transgene expresses the transcription factor Gal4 in a DAL-specific anatomical pattern. The Gal4 protein acts on UAS sequences to make the FLP recombinase (second line cartoon). The third transgene contains a fragment from the *period* promoter (*per*P) and a FLP-out cassette (triangles flanking mCherry, followed by stop codons in all reading frames) upstream of the coding region for luciferase (missing its ATG start codon). The black arrow signifies the start of transcription and translation. In the presence of FLP, recombination at the FRT sites results in deletion of one FRT site and the intervening sequences, including the translation termination codons (fourth line cartoon). **(B)** FLP expression in DAL neurons results in oscillating reporter activity. When the LABL system is driven using the DAL-specific Gal4 lines (G0338 [left plot in orange] or G0431 [right plot in magenta]), transcriptional activity oscillates. Luminescence (in arbitrary units) is plotted as a function of time. Daytime and nighttime periods are shown in light and dark gray and the vertical black lines indicate a 24-h period. The best fit maxima and minima for each 24-h period are shown with circles. **(C)** LABL oscillates with an approximate 24-h periodicity in DAL neurons. Data from the LABL experiments (*n* = 4) is analyzed as before (Johnstone et al., 2022). Morlet wave analysis for the entire 9 days shows that the periodicity of both reporters is about 24 h. Confidence intervals are shown in shades of color and indicated above the figure. The violin plots indicate the spread of all the data points. **(D)** Mutations in *tim* and *cyc* do not significantly affect 3d memory. The 3d performance indices for wild-type (*iso31*) and mutant stocks are plotted as a function of genotype. There is no significant difference in either mutant when compared to its wild type control.

The name of a fly stock (G0388) is incorrect, and has been corrected to G0338 throughout the article.

A correction has been made to the section Materials and methods, *Flies*, first paragraph:

“All the flies used have been validated and published previously. For the reporter experiments, the G0338-or G0431-Gal4 driver lines (which are both expressed in DAL neurons) are combined with the two other transgenes (*period-*promoter-FRT-*luciferase* and 20xUAS-FLP) that comprise the LABL system (Chen et al., 2012; Lin et al., 2021; Johnstone et al., 2022). For olfactory behavior, circadian and sleep experiments, progeny from a cross between one parent homozygous for two different transgenes and a second parent homozygous for a third transgene are used. The DAL specific driver line (G0431) is combined with the *tubulin*P-Gal80ts transgene in the doubly transgenic parent (*w*^1118^; G0431-Gal4; *tubulin*P-GAL80^ts^). For the rest of this report, this genotype will be shortened to G0431; *tubulin*P-Gal80^ts^. The other parent is one of: [*w*^1118^; +/+; UAS-*clk*^Δ^ (Tanoue et al., 2004)], [*w*^1118^; UAS-*cyc*^Δ^; +/+ (Tanoue et al., 2004)], [*w*^1118^; UAS-*clk*^RNAi^; +/+ (VDRC 107576)] or [*w*^1118^; UAS-*cyc*^RNAi^; +/+ (VDRC 110455)]. These four genotypes will be shortened to: UAS-*clk*^Δ^, UAS-*cyc*^Δ^, UAS-*clk*^RNAi^ and UAS-*cy*c^RNAi^. The *tubulin*P-Gal80^ts^ transgene makes the Gal80^ts^ protein ubiquitously. In the progeny flies that contain all three transgenes, at low temperature (20 °C) the Gal80^ts^ protein is active and represses Gal4 from acting. At the restrictive temperature (29 °C), the Gal80^ts^ protein is inactive and Gal4 becomes active. For assays of circadian locomotor activity and sleep, the parents and progeny from two of the crosses (those involving UAS-*clk*^Δ^ _or UAS-*cyc*^Δ^) are used. The UAS-*cyc*^Δ^ *_*and UAS-*clk*^Δ^ *_*flies are from P. Hardin, although the UAS-*clk*^Δ^ stock is available through BDSC (#36319). The *cyc*°, *tim*° stocks along with their isogenic wild-type control flies are from A. Sehgal. The G0338 and G0431-Gal4 driver lines and the doubly transgenic parent (G0431; *tubulin*P-GAL80^ts^) are from T. Tully.”

A correction has been made to the section Materials and methods, *LABL reporter assay*:

“Flies of the correct genotype (with the DAL-specific Gal4 driver lines [either G0338 or G0431], the LABL transgenic reporter and the UAS-FLP transgene) are assayed and analyzed as described previously (Johnstone et al., 2022).”

Corrections have been made to the section Results, A *period*-promoter based reporter cycles in DAL neurons, second and third paragraphs:

“Initially we use the LABL reporter system to ask if there is detectable oscillatory transcriptional activity in DAL neurons (Johnstone et al., 2022). Reporter activity is an indirect measure for CLK and CYC activity since the LABL reporter contains a minimal *period* promoter that results in oscillatory activity if CLK and CYC bind to a consensus E-box sequence (Bargiello et al., 1987). Figure 1A cartoons the necessary components in this system, and we test it in DAL using the G0338 or G0431 Gal4 driver lines (Chen et al., 2012).

Figure 1B shows reporter activity when FLP is expressed in DAL neurons. Luminescence (in arbitrary units) is plotted as a function of time for the G0338- (left side plot in orange) and G0431-driven (right side plot in purple) reporters, with the subjective day (light gray) and subjective night (dark gray) periods indicated. The closed circles represent the best fits for maxima and minima of the oscillating activity each day. Although the amplitudes are low, both G0338-and G0431-driven reporter activities oscillate. Morlet wavelet analysis (Figure 1C) indicates that the periodicity of the oscillations cluster around 24 h for all confidence intervals, consistent with circadian-regulated transcription. The violin plots to the right of each Morlet wavelet analysis show that the periodicities across the entire 9d interval cluster around 24 h for reporters activated using both drivers. Since CLK/CYC heterodimers are major contributors to oscillating transcription from the *period* promoter, they are likely to be expressed and active in DAL neurons, confirming findings from prior work (Bargiello et al., 1987; Chen et al., 2012; Crocker et al., 2016). Crocker et al. reported detecting the expression of the circadian genes *cyc, pdp1* and *cry* in DAL neurons using a scRNA sequencing approach (Crocker et al., 2016).”

The original version of this article has been updated.

