# Supplementary figures and images for: The circadian genes are required in DAL neurons for Drosophila long-term memory formation
Source: Front Neurosci. 2025 Jun 30;19:1623251. doi: 10.3389/fnins.2025.1623251 (PMC12256545; doi:10.3389/fnins.2025.1623251)

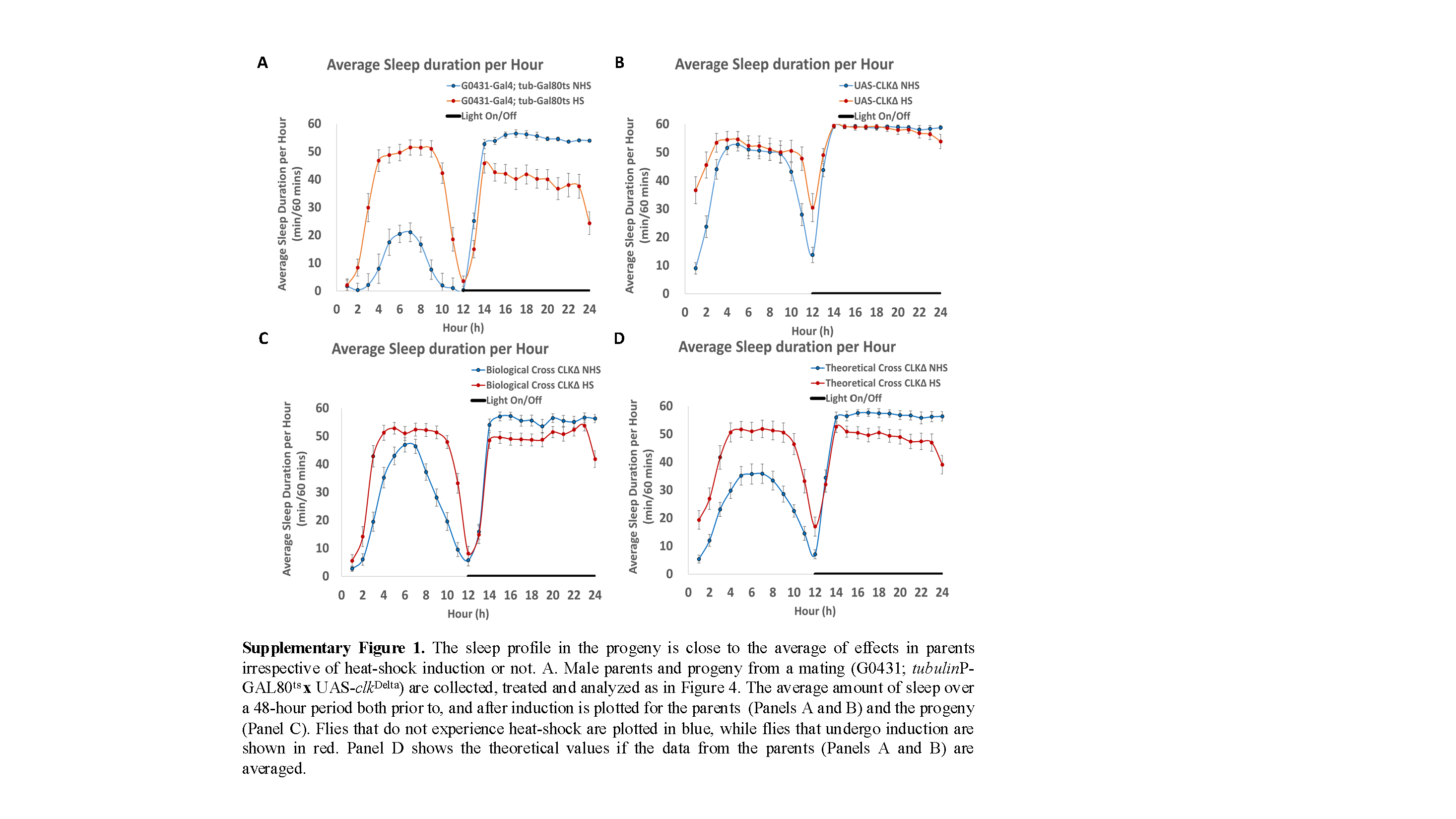

Supplement: Supplementary file 1 [file Image_1.tiff]

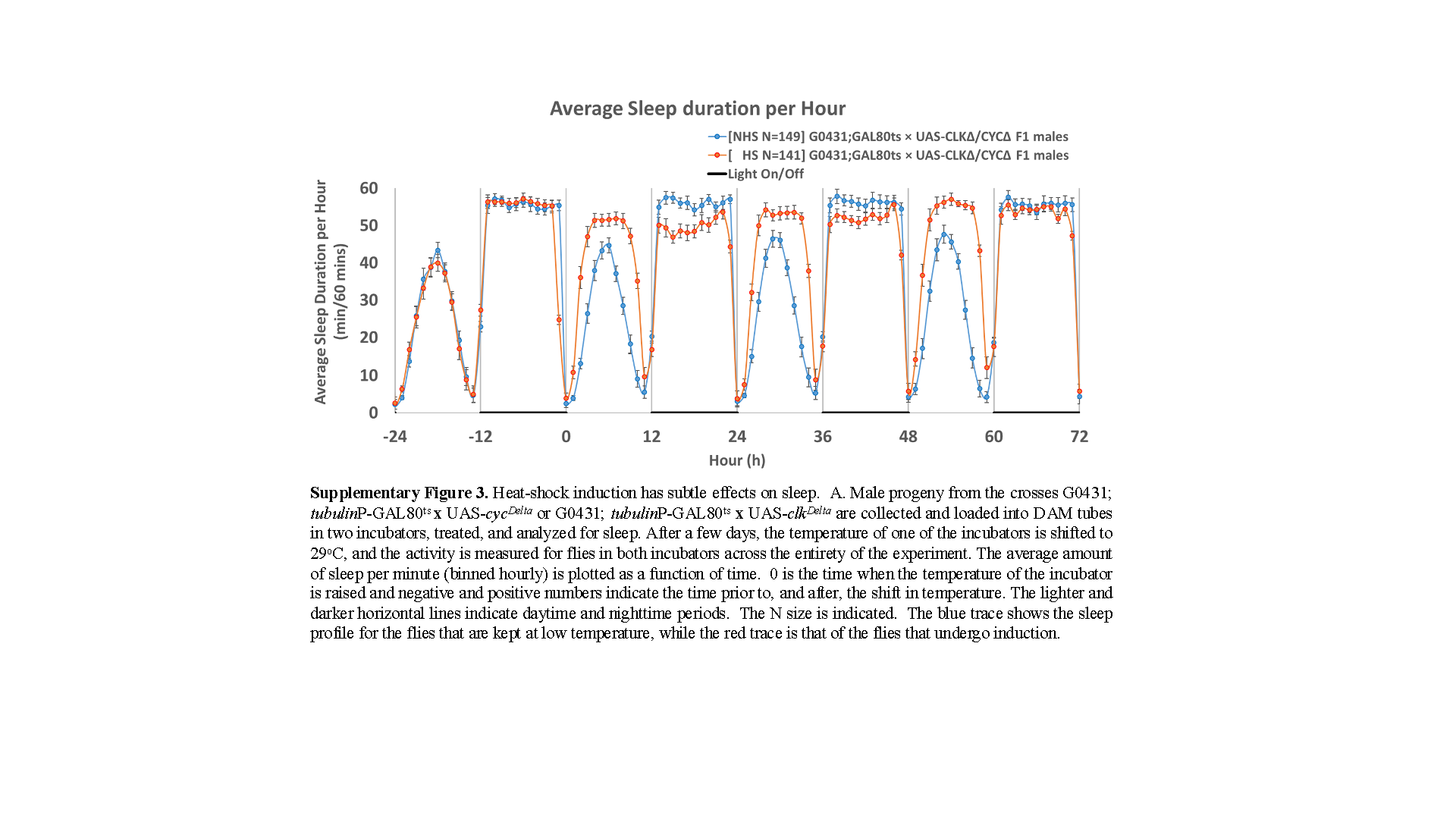

Supplement: Supplementary file 3 [file Image_3.tiff]
