# Supplementary material for: The circadian genes are required in DAL neurons for Drosophila long-term memory formation
Source: Front Neurosci. 2025 Jun 30;19:1623251. doi: 10.3389/fnins.2025.1623251 (PMC12256545; doi:10.3389/fnins.2025.1623251)
Supplement: Supplementary file 2 [file Image_2.pdf]

Sleep	Total			Daytime			Nighttime		
	UAS-CLK x G0431;GAL80ts	Pct %	p-value	UAS-CLK x G0431;GAL80ts	Pct %	p-value	UAS-CLK x G0431;GAL80ts	Pct %	p-value
Pre-HS<br>Post-HS	952<br>1021	107%	0.000	314<br>463	147%	0.000	637<br>558	88%	0.000
Bout Number	Total			Daytime			Nighttime		
	UAS-CLK x G0431;GAL80ts	Pct %	p-value	UAS-CLK x G0431;GAL80ts	Pct %	p-value	UAS-CLK x G0431;GAL80ts	Pct %	p-value
Pre-HS<br>Post-HS	18.6<br>33.3	179%	0.000	12.3<br>17.9	145%	0.000	6.32<br>15.4	244%	0.000
Bout Length	Total			Daytime			Nighttime		
	UAS-CLK x G0431;GAL80ts	Pct %	p-value	UAS-CLK x G0431;GAL80ts	Pct %	p-value	UAS-CLK x G0431;GAL80ts	Pct %	p-value
Pre-HS<br>Post-HS	61.5<br>35.4	58%	0.000	30.6<br>36.9	121%	0.023	212<br>49.9	23%	0.000

Sleep	Total			Daytime			Nighttime		
	UAS-CYC x G0431;GAL80ts	Pct %	p-value	UAS-CYC x G0431;GAL80ts	Pct %	p-value	UAS-CYC x G0431;GAL80ts	Pct %	p-value
Pre-HS<br>Post-HS	847<br>1028	121%	0.000	208<br>442	212%	0.000	639<br>586	92%	0.000
Bout Number	Total			Daytime			Nighttime		
	UAS-CYC x G0431;GAL80ts	Pct %	p-value	UAS-CYC x G0431;GAL80ts	Pct %	p-value	UAS-CYC x G0431;GAL80ts	Pct %	p-value
Pre-HS<br>Post-HS	15.9<br>25.6	160%	0.000	8.75<br>14.9	171%	0.000	7.19<br>10.6	148%	0.000
Bout Length	Total			Daytime			Nighttime		
	UAS-CYC x G0431;GAL80ts	Pct %	p-value	UAS-CYC x G0431;GAL80ts	Pct %	p-value	UAS-CYC x G0431;GAL80ts	Pct %	p-value
Pre-HS<br>Post-HS	70.0<br>47.7	68%	0.000	29.5<br>43.3	147%	0.001	193<br>78.7	41%	0.000

Sleep	Total			Daytime			Nighttime		
	G0431;GAL80ts	Pct %	p-value	G0431;GAL80ts	Pct %	p-value	G0431;GAL80ts	Pct %	p-value
Pre-HS<br>Post-HS	726<br>848	117%	0.000	99<br>404	408%	0.000	591<br>444	75%	0.00
Bout Number	Total			Daytime			Nighttime		
	G0431;GAL80ts	Pct %	p-value	G0431;GAL80ts	Pct %	p-value	G0431;GAL80ts	Pct %	p-value
Pre-HS<br>Post-HS	16.0<br>34.2	215%	0.000	7.60<br>15.1	198%	0.000	8.13<br>19.1	235%	0.000
Bout Length	Total			Daytime			Nighttime		
	G0431;GAL80ts	Pct %	p-value	G0431;GAL80ts	Pct %	p-value	G0431;GAL80ts	Pct %	p-value
Pre-HS<br>Post-HS	146<br>58.7	40%	0.000	12.7<br>32.1	235%	0.000	138<br>26.6	19%	0.000

Sleep	Total			Daytime			Nighttime		
	UAS-CLK	Pct %	p-value	UAS-CLK	Pct %	p-value	UAS-CLK	Pct %	p-value
Pre-HS<br>Post-HS	1158<br>1248	108%	0.000	467<br>562	120%	0.000	691<br>686	99%	0.329
Bout Number	Total			Daytime			Nighttime		
	UAS-CLK	Pct %	p-value	UAS-CLK	Pct %	p-value	UAS-CLK	Pct %	p-value
Pre-HS<br>Post-HS	27.3<br>20.5	75%	0.000	21.5<br>13.7	64%	0.000	5.87<br>6.79	116%	0.211
Bout Length	Total			Daytime			Nighttime		
	UAS-CLK	Pct %	p-value	UAS-CLK	Pct %	p-value	UAS-CLK	Pct %	p-value
Pre-HS<br>Post-HS	258<br>107	41%	0.000	26.5<br>89.4	338%	0.000	232<br>264	114%	0.260

Sleep	Total			Daytime			Nighttime		
	UAS-CYC	Pct %	p-value	UAS-CYC	Pct %	p-value	UAS-CYC	Pct %	p-value
Pre-HS<br>Post-HS	1033<br>1215	118%	0.000	358<br>519	145%	0.000	675<br>696	103%	0.000
Bout Number	Total			Daytime			Nighttime		
	UAS-CYC	Pct %	p-value	UAS-CYC	Pct %	p-value	UAS-CYC	Pct %	p-value
Pre-HS<br>Post-HS	19.9<br>13.1	66%	0.000	14.5<br>9.90	68%	0.000	5.44<br>3.23	59%	0.000
Bout Length	Total			Daytime			Nighttime		
	UAS-CYC	Pct %	p-value	UAS-CYC	Pct %	p-value	UAS-CYC	Pct %	p-value
Pre-HS<br>Post-HS	63.7<br>138	217%	0.000	31.1<br>80.4	258%	0.000	309<br>452	147%	0.000

Supplementary Figure 2. Quantitative analysis of sleep for the parents and progeny stocks. Sleep analysis is done on male parents and progeny from two matings (G0431; *tubulin*P-GAL80^{ts} **x** UAS-*clk*^{Delta} and G0431; *tubulin*P-GAL80^{ts} **x** UAS-*cyc*^{Delta}) both pre- and post-induction. The average sleep for 48-hour periods both before and after induction is measured and analyzed. The percentage change (post-induction/pre-induction) and its significance is shown. The N size for the UAS-*clk*^{D} progeny =77 and for the UAS-*cyc*^{D} progeny =64. The N size for the parents alone: G0431; *tubulin*P-GAL80^{ts} =54, for UAS-*clk*^{D} =62, and for UAS-*cyc*^{D} =64. For significance, 0.00 represents $p<0.01$ and 0.000 represents $p<0.001$.
